# Shark-based tourism presents opportunities for facultative dietary shift in coral reef fish

**DOI:** 10.1371/journal.pone.0221781

**Published:** 2019-08-29

**Authors:** Joshua A. Drew, Mallory McKeon

**Affiliations:** Columbia University, Department of Ecology, Evolution, and Environmental Biology, New York, NY United States of America; Florida Institute of Technology, UNITED STATES

## Abstract

Tourism represents an important opportunity to provide sustainable funding for many ecosystems, including marine systems. Tourism that is reliant on aggregating predator species in a specific area using food provisioning raises questions about the long-term ecological impacts to the ecosystem at large? Here, using opportunistically collected video footage, we document that 61 different species of fish across 16 families are consuming tuna flesh at two separate shark dive tourism operations in the Republic of Fiji. Of these fish, we have resolved 55 to species level. Notably, 35 (63%) of the identified species we observed consuming tuna flesh were from ostensibly non-piscivorous fishes, including four Acanthuridae species, a group primarily recognized as browsers or grazers of algae and epibenthic detritus. Our results indicate that shark diving is having a direct impact on species other than sharks and that many species are facultatively expanding their trophic niches to accommodate the hyperabundance of resources provided by ecotourism.

## Introduction

Tourism has been long suggested as a mechanism for supporting sustainable use of protected ecosystems, particularly in marine areas [1]. One form of ecotourism that has become popular in the last decade is the use of large Elasmobranches (sharks and rays) as foci for dive tourism [2–5]. One prominent question regarding this activity is: what are the impacts of dive tourism on the local ecology? This question becomes particularly pertinent in sites where food is used to help aggregate charismatic megafauna for tourists. At the community level, studies have shown changes in diversity and mean trophic level in shark feeding in some areas, [4] and a reduction in benthic diversity in others [6]. A parallel question remains regarding population-level impacts of this trophic supplementation: what, if any, changes in trophic functional groups occur during these predictably occurring, and frequent, times of trophic hyperabundance?

Although functional groups are a useful concept in ecology, the rigidly defined dietary boundaries for different functional groups do not adequately acknowledge the potential for plasticity that many species exhibit under certain circumstances. For example, in response to community and/or population level processes, some species opportunistically expand their expected trophic envelopes [7,8]. In marine systems, there are numerous observations of prey expansion as a function of prey availability in species ranging from copepods (*Calanus pacificus*; [9]) to orca (*Orcinus orca*; [10]). In terrestrial systems, novel resource availability can also lead to opportunistic trophic expansion. Snowshoe hares (*Lepus americanus*) have been observed scavenging on mammalian and avian prey [11] while eastern cottontail rabbits (*Sylvilagus floridanus*) have been recorded scavenging avian prey [12].

Functional groups may also blur during periods of resource hyperabundance. On land, Alaskan beaver (*Castor canadensis*) feed on chinook salmon (*Oncorhynchus tshawytscha*) carcasses during spawning aggregations [13]. In reef systems, numerous invertebrates (scleractinian corals, echinoderms and bivalves) coordinate mass spawning in both predictable and unpredictable events. These mass spawning events can provide a food source for reef fish, where numerous species aggregate to feed on nutrient-rich gametes [14].

In these examples, the trophic expansion is a response to an ephemeral resource which is characterized by three primary characteristics: (1) predictable in time, (2) short-lived, and (3) annual in periodicity. Food provisioning through tourism diverges from natural hyperabudnace in its periodicity, with food provisioning often occurring on a weekly or even daily basis.

In some ways, commercial shark feeding mimics natural exploitation of hyperabundant resources [15] as pelagic fish (tuna heads) are fed to the sharks, thus providing a human-facilitated trophic linkage between pelagic and near-shore systems. In fact, while a recent isotopic study suggested that trophic supplementation to bull sharks (*Carcharhinus leucas*) from tuna heads represented only a minor component of their diet [16], a bioenergetics model suggested that at least some sharks could meet or exceed their daily dietary requirements solely from provisioned food [17]. Thus at least on local time scales, tourism-based provisioning can provide an important role in the population ecology of reef apex predators.

While the commercial draw of shark feeding is the sharks themselves, the ecological impact of food provisioning on the diet and behavior of the lower trophic-level fishes found within the shark feeding sites remains an open area of investigation. Here, we take a first step at resolving this question by reporting the diversity of fishes that were observed directly feeding upon human-facilitated trophic supplements at two shark-based ecotourism feeding areas in the Republic of Fiji. In doing so, we present evidence for the direct trophic supplementation by a commercial tourism operation of coral reef fishes across multiple functional groups and demonstrate that many of these fishes facultatively shift their diet despite ostensibly being consumers. Our work suggests that the resource subsidies created by shark-diving could percolate throughout the reef fish community and that these fish are drawing upon latent behavioral plasticity to expand their traditional trophic levels in response to anthropogenic hyperabundance occurring on daily time scales.

## Methods

We opportunistically collected observational data in Beqa Lagoon (18°24′S, 178°08′E), off the island of Viti Levu in the Republic of Fiji. The area is a no-take marine protected area (MPA) where two companies, Beqa Adventure Divers (BAD) and AquaTrek, have established shark-based diving programs within the MPA.

**Fig 1:**
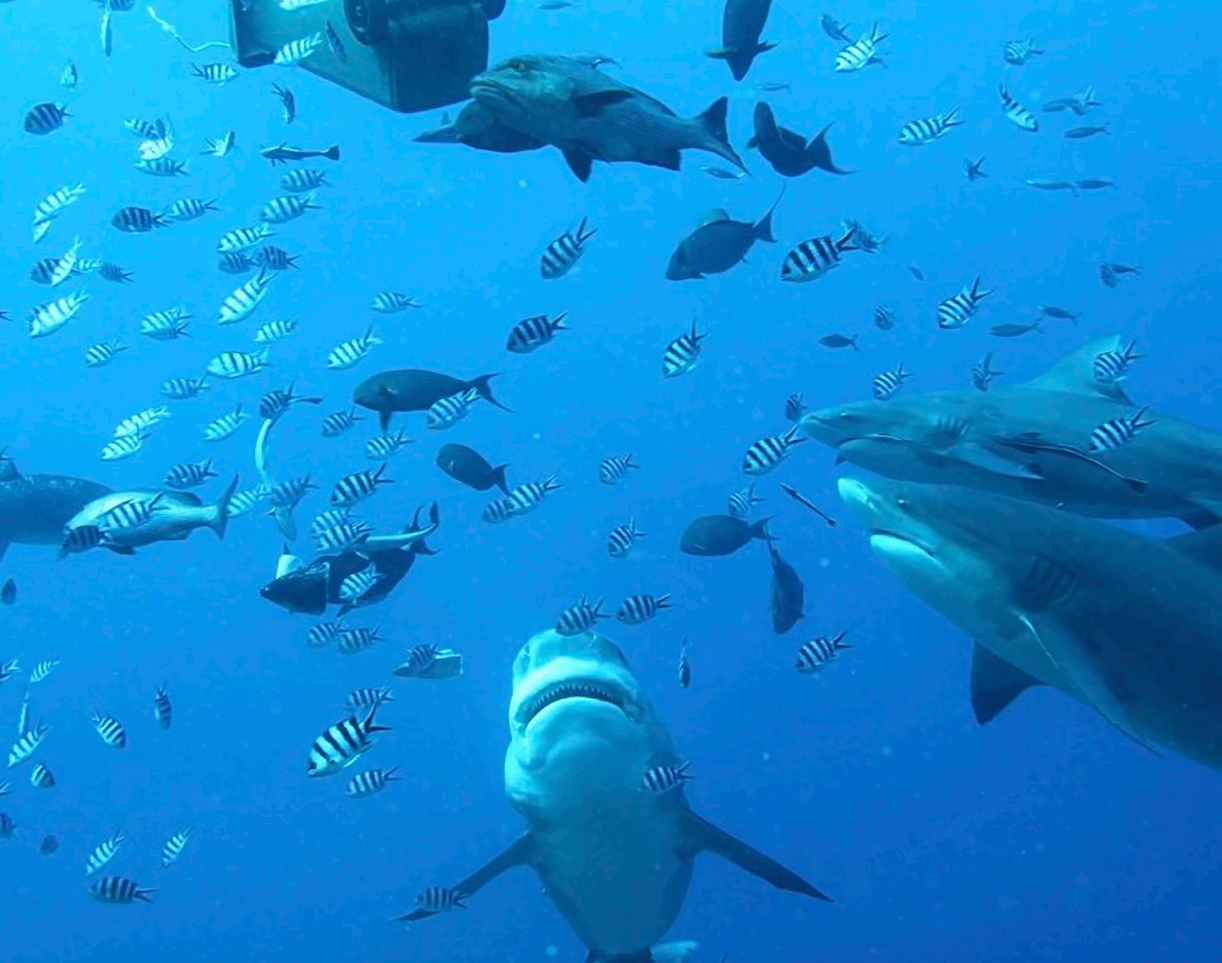
A large female bull shark, *Carcharhinus lecuas* approaches divers waiting to be fed. The crowd of small fish includes piscivores (*Lutjanus bohar*) herbivores (*Acanthurus xanthopterus*), and planktivores (*Abudefduf sexfasciatus*) is gathering to feed.

Because these observations were made as part of commercial dives, there were no additional permissions necessary. Additionally, there were no permits required to collect observational data on any endangered or protected species present. The dives take place six days a week, year-round, at a coral rubble area approximately 20 m deep. Each trip consists of two tank dives for about 10–15 tourists each. The main attraction of these dives is the hand feeding of tuna heads (typically *Thunnus obesus* and *Katsuwonus pelamis*) to sharks, mostly bull sharks (*Carcharhinus leucas*) with occasional tiger (*Galeocerdo cuvier*) and sickle-fin lemon (*Negaprion acutidens*) sharks. The two organizations differ in that AquaTrek practices the dissemination of chum as an attractant before and during feeding events, while BAD exclusively hand feeds sharks. The sharks then consume fish heads while swimming past the customers, often passing within about 2 m from the tourists. Trained dive masters are stationed above the tourists and across the feeding arena to maintain safety measures. As sharks consume the heads, they create small flakes of tuna suspended in the water column providing a locally hyperabundant, temporally predictable, resource for other fish who swarm around and behind the shark (**Fig 1**).

Aquatrek has incorporated non-selective feeding methods and therefore feeds a broader spectrum of sharks and other predatory fish species, such as grouper, with limited discretion [4]. AquaTrek also feeds a number of species that are excluded from direct feeding at BAD [18], including black-tipped (*C*. *melanopterus*), white-tipped (*Triaenodon obesus*) and tawny nurse (*Nebrius ferrugineus*) sharks. The non-shark component of the community at both sites is mostly consistent and is composed of a suite of Indo-Pacific fishes commonly seen in Fiji (see http://fijisharkdive.com/conservation/shark-reef-fish-list/ for a list of species).

Our data consisted of opportunistically filmed shark feeding events that took place during July of 2016. During this time, one diver (MGM) recorded 29 minutes of video footage across three dives at AquaTrek, and 9 minutes of video footage across two dives at BAD, which was supplemented by 7 min of footage from 2012 shot by JAD. For each recording, we identified the species observed participating in feeding events. Video footage was reviewed after in-person observations of facultative consumption of food scraps by non-shark species. All fish species present and active at feeding events were identified to the lowest possible taxonomic level. We used an on-line database, FishBase [19] to query average trophic level, dietary composition, and standard length for all species.

## Results

We observed a total of 61 different species of fish from 18 families directly consuming tuna pieces in the water column. Because of the schooling nature of many of these species, it was not feasible to calculate the number of individual observations. Of the 61 observed species, 55 were identified to the species level; of those 55, 35 (63.6%) were species reported as non-piscivorous or scavengers, with 20 (36%) being primarily fish eaters, 14 (25%) being listed as planktivores and 4 (7%) being listed as primary algae or detritus eaters (**Table 1**). The sizes of the fish consuming tuna cover a wide spectrum, ranging from small (the 12.5 cm maximum standard length butterflyfish, *Chaetodon mertensii*) to massive (the 750 cm maximum length tiger shark, *Galeocerdocuvier*), suggesting that larger sharks are not competitively excluding smaller fishes the way congeners are excluded at some sites [18]. The average trophic level for all species observed was 3.47 (N = 55, standard deviation = 0.63), indicating that most species were between secondary and tertiary consumers. When we examined the species that were not considered non-scavenging piscivorous in the literature, the average fell to 3.1 (N = 38, standard deviation = 0.548).

**Table 1:** Fishes observed taking bites of tuna at each site, and characteristic diet associated with each species. For AquaTrek and Beqa a “1” signifies that a species was observed at that particular location, while a “0” indicates that species was not observed during our sampling. Typical food, average trophic level and maximum standard length were taken from FishBase and the literature.

Species	Family	AquaTrek	Beqa	Typical food	Maximum Length (CM)	Trophic Level
*Acanthurus sp*.	*Acanthuridae*	0	1	-	-	-
*Acanthurus nigricauda*	*Acanthuridae*	1	0	Detritus/algae	40	3
*Acanthurus olivaceus*	*Acanthuridae*	1	0	Detritus/algae	35	2.2
*Ctenochaetus striatus*	*Acanthuridae*	1	1	Detritus/algae	26	2
*Balistoides conspicillum*	*Balistidae*	1	0	Crustaceans	50	3.3
*Caesio caerulaurea*	*Caesionidae*	1	1	Plankton	35	3.4
*Caesio teres*	*Caesionidae*	1	0	Plankton	40	3.4
*Caesio varilineata*	*Caesionidae*	0	1	Plankton	40	3.4
*Pterocaesio digramma*	*Caesionidae*	1	0	Plankton	30	3.4
*Pterocaesio tile*	*Caesionidae*	0	1	Plankton	30	3.3
*Pterocaesio trilineata*	*Caesionidae*	1	0	Plankton	20	3.4
*Caranx ignobilis*	*Carangidae*	1	0	Fish	60	4.2
*Caranx lugubris*	*Carangidae*	1	0	Fish	100	4.5
*Carcharhinus leucas*	*Carcharhinidae*	1	1	Fish	360	4.4
*Negaprion acutidens*	*Carcharhinidae*	1	0	Fish	380	4.4
*Galeocerdo cuvier*	*Carcharhinidae*	1	0	Fish	750	4.5
*Chaetodon sp*.	*Chaetodontidae*	1	1	-	-	-
*Forcipiger flavissimus*	*Chaetodontidae*	1	0	Benthic invertebrates	22	3.1
*Heniochus monoceros*	*Chaetodontidae*	1	0	Benthic invertebrates	24	3.5
*Chaetodon mertensii*	*Chaetodontidae*	1	0	Coral polyps	12.5	3
*Chaetodon auriga*	*Chaetodontidae*	1	0	Coral Polyps/Invertebrates	16	3.3
*Chaetodon kleinii*	*Chaetodontidae*	0	1	Coral Polyps/Invertebrates	15	3.1
*Chaetodon ulietensis*	*Chaetodontidae*	1	0	Coral Polyps/Invertebrates	15	2.7
*Heniochus acuminatus*	*Chaetodontidae*	1	1	Zooplankton/Benthic invertebrates	25	3.5
*Paracirrhites forsteri *	*Cirrhitidae*			Fishes and invertebrates	22	4.3
*Echeneis naucrates*	*Echeneidae*	0	1	Fish	110	3.5
*Remora remora*	*Echeneidae*	1	1	Fish	86	3.5
*Nebrius ferrugineus*	*Ginglymostomatidae*	1	0	Fish	320	4.1
*Myripristis sp*.	*Holocentridae*	1	0	-	-	-
*Anampses caeruleopunctatus*	*Labridae*	1	0	Benthic invertebrates	42	3.3
*Chlorurus bleekeri*	*Labridae*	1	0	Algae	49	2
*Thalassoma lunare*	*Labridae*	1	1	Benthic invertebrates	45	3.5
*Thalassoma lutescens*	*Labridae*	0	1	Benthic invertebrates	30	3.7
*Cheilinus trilobatus*	*Labridae*	0	1	Sea urchins	45	3.9
*Labroides bicolor*	*Labridae*	1	1	Ectoparasites	15	4
*Scarus sp*.	*Labridae*	1	0	-	-	-
*Lethrinus nebulosus*	*Lethrinidae*	1	0	Benthic invertebrates	87	3.6
*Lutjanus russelli*	*Lutjanidae*	1	1	Benthic invertebrates	50	3.9
*Lutjanus monostigma*	*Lutjanidae*	1	0	Fishes and benthic crustaceans	60	4.3
*Lutjanus gibbus*	*Lutjanidae*	1	1	Fishes and invertebrates	50	3.8
*Lutjanus quinquelineatus*	*Lutjanidae*	0	1	Fishes and invertebrates	38	3.7
*Lutjanus semicinctus*	*Lutjanidae*	1	0	Fishes and invertebrates	35	4.2
*Macolor niger*	*Lutjanidae*	1	0	Fishes and invertebrates	75	4
*Lutjanus fulvus*	*Lutjanidae*	1	0	Fishes, shrimp and cephalopods	40	3.6
*Lutjanus kasmira*	*Lutjanidae*	1	1	Fishes, shrimp and crustaceans	40	3.9
*Lutjanus bohar*	*Lutjanidae*	1	1	Small fish and invertebrates	90	4.3
*Lutjanus ehrenbergii*	*Lutjanidae*	1	0	Small fish and invertebrates	35	3.9
*Parupeneus crassilabris*	*Mullidae*	1	0	Benthic invertebrates	38	3.6
*Parupeneus insularis*	*Mullidae*	1	0	Crustaceans	30	3.7
*Pomacentrus sp*.	*Pomacentridae*	1	0	-	-	-
*Abudefduf vaigiensis*	*Pomacentridae*	0	1	Plankton	20	2.6
*Abudefduf sexfasciatus*	*Pomacentridae*	1	1	Plankton	19	2.4
*Amblyglyphidodon aureus*	*Pomacentridae*	1	0	Plankton	13	2.7
*Chromis alpha*	*Pomacentridae*	1	0	Plankton	8.5	3
*Chromis elerae*	*Pomacentridae*	0	1	Plankton	7	2.7
*Chromis leucura*	*Pomacentridae*	1	0	Plankton	8.5	3.1
*Chromis margaritifer*	*Pomacentridae*	1	0	Plankton	9	3
*Dacyllus flavicaulus*	*Pomacentridae*	1	1	Plankton	10	3.3
*Epinephelus sp*.	*Serranidae*	1	0	-	-	-
*Aethaloperca rogaa*	*Serranidae*	1	1	Fish	60	4.2
*Epinephelus lanceolatus*	*Serranidae*	1	0	Fish	270	4

## Discussion and conclusions

Despite wide variations in morphology, the teeth of large sharks effectively puncture and slice flesh [20,21]. However, due to sharks’ lack of lips and general feeding habits, small pieces of tuna fall out of the sharks' mouths, entering the water column for smaller fish species to eat.

Our observations demonstrate that a variety of common coral reef fish facultatively supplement their regular diet with food as an unintended byproduct of shark provisioning activity. Moreover, this dietary flexibility appears to be widespread both phylogenetically and functionally, with fish from multiple families, and numerous functional groups exhibiting such shifts. This ability to expand their niche occurs because of a spatially and temporally predictable resource pulse mediated through tourism and the biomechanics of shark feeding. Thus, under certain circumstances, functional groups and measurements of average trophic level may not fully capture the diversity of food consumed by these fishes.

Many of the species we observed already exhibit some dietary flexibility, particularly in light of temporally or spatially ephemeral food resources, suggesting that there is an inherent behavioral template that is being expanded upon within dive sites. For example, the planktivorous damselfish *Abudefduf sexfasciatus* (Pomacentridae) typically feed on zooplankton [22,23]. However, during coral spawning events, they shift to fat and protein-rich coral gametes [24]. We frequently observed schools of *A*. *sexfasciatus* chase after scraps of flesh when sharks were fed tuna heads. Similarly, multiple members of the planktivorous Caeseionidae were found consuming small particles of tuna despite their diets usually consisting of pelagic crustaceans [14].

In addition, fish that typically focus on benthic invertebrates, including the coral-eating Chaetodonitdae (here represented by *Chaetodon mertensii*, *C*. *kleinii* and *C*. *auriga*), were able to expand their diets. Notably, these three fish on occasion demonstrate a more generalist nature, consuming and successfully digesting a number of food sources outside of their typical scleractinian invertebrates [25, 26].

Other mobile benthic invertebrate predators that we observed feeding on tuna have demonstrated dietary flexibility in the wild. *Forcipiger flavissimus* (Chaetodontidae) normally feed on small benthic invertebrates living interstitially within coral heads [27] but can switch to cleaning ectoparasites off of other fish under certain circumstances [28]. Similarly, the benthic invertebrate consumer *Thalassoma lunare* (Labridae) will seasonally switch to piscine prey, consuming a large number of fish recruits [29, 30]; some aggregations have even been known to consume coral spawn [24].

Other species of fish observed consuming tuna in the water were more surprising given their physiological or behavioral limits. For example, the Acanthurid, *Ctenochaetus striatus* has very fine, comb-like teeth (the genus name translates to “bristle tooth”) and a gut microflora that effectively digests algae and benthic detritus [31, 32]. Here we observed individuals from this and other confamilial species in the water column consuming fish pieces (Supplemental 1). Analysis of the gut contents of *C*. *striatus* and *Acanthurus olivaceus* by Choat et al. [33] suggested that while the species appears to be feeding primarily on algae, the majority of their diet actually consisted of organic detritus, thus indicating that they maintain at least some physiological mechanism to digest a diversity of non-plant material.

Lastly, the cleaner wrasse (*Labroides bicolor* Labridae), a roving obligate cleaner which consumes both ectoparasites and fish mucus [34], was observed consuming fish material in the water column. Cleaning fish are usually constrained to coral outcroppings, where they solicit client fish. Therefore, extended voyages into the water column to capture free-floating flesh is a surprising choice, as it would expose them to a greater risk of predation. However, at the shark dive site, lack of predation threat allows for niche expansion—cleaner wrasses are more likely to take non-preferential food in the presence of predatory fish [35].

Where our study differs from those listed above is that in the aforementioned cases the periods of resource abundances were naturally occurring and temporally limited. Here we see that the evolutionally and behaviorally plastic ability to respond to periods of resource hyperabudance can be applied to Anthropocene settings (in this case, a daily pulse of tuna heads placed in the water to provide tourists’ entertainment). The niche variation hypothesis [36] suggests that generalist populations will expand their niches under release from interspecific competition. Given that observed predation rates are low (pers. obsv.) and food availability is high (e.g. tuna in the water) these populations of generalist feeders appear to be expanding their niches to include human-mediated resources.

Our work represents an important first step in understanding how the ecology of coral reefs is impacted by tourism. Stable isotope studies have suggested that food provisioning at Beqa shark dive sites could meet daily nutritional diets for some bull sharks [17] Whether the tuna subsidy to smaller, opportunistically feeding species is energetically significant for other species remains an open question.

Our data were collected opportunistically, are observational by nature, and were limited by the dangers inherent in conducting field research in the presence of large, actively feeding sharks. As all observations were made from a safe recording distance, we were unable to definitively quantify the full extent to which allochthonous subsidies are percolating throughout these reef systems. It is possible that our results underestimate the scope of this trickle-down ecology and that small, cryptic fishes such as blennies, gobies, and other benthic dwellers may also be receiving supplementation, either directly or indirectly.

Our work here highlights the potential ecological impact of anthropogenically derived food supplementation on the daily energy budget of lower trophic level reef fish. In doing so, our research opens up several potentially interesting lines of further investigation. For example, what are the behavioral impacts of typically bottom-dwelling fish now feeding higher in the water column and the concordant potential shifts territoriality within and among species? For sex-changing species, resource availability helps drive mating strategy [37] and thus the reproductive ecology of several of these species could potentially differ because of food provisioning. Lastly, and importantly, observations of feeding do not mean *per se* that supplemental food is a significant component to the overall energy budget of these species. Future investigations using stable isotopes coupled with bite calculations should be carried out to determine if the excess food from shark provisioning is a major component of, or an ephemeral supplement to, their daily dietary budget. The continued proliferation of shark-based tourism and the ecological importance of this trophic supplementation increase the relevance of this topic. Moreover, these observations contribute to a larger body of research considering the ecological impacts of resource pulses, behavior flexibility, and the dynamics of functional groups within the Anthropocene.

## Supporting information

S1 Fig A video showing a piece of tuna in the water column at Beqa Fiji. The tuna entered the water column from the top of the image, where it was immediately surrounded by several nominally herbivorous *Acanthurus xanthopterus*, which were observed consuming the tuna flesh. The tuna head then drew attention from several piscivorous *Lutjanus bohar*. At 4 seconds a large *Carcharhinus leucas* frightens off other fish, and consumed the tuna head whole. Throughout the video the fish are surrounded by a school of planktivorous *Abudefduf sexfasciatus*.
(GIF)

S2 Fig A swarm of fish surround the shark feeders at BAD. Note the aggregation following the *C*. *lucas* after it takes the tuna head, followed by another aggregation of smaller fish trying to get into the tuna holding container.
(MOV)

S3 Fig An aggregation of fish greets the diver carrying the tuna head. Once opened several remora (*Remora remora*) and tangs (Acanturus sp.) break away to feed on a tuna head, while a snaller (Lutanus sp.) looks for an opening.
(MOV)
