# Supplementary figures and images for: Shark-based tourism presents opportunities for facultative dietary shift in coral reef fish
Source: PLoS One. 2019 Aug 29;14(8):e0221781. doi: 10.1371/journal.pone.0221781 (PMC6715274; doi:10.1371/journal.pone.0221781)

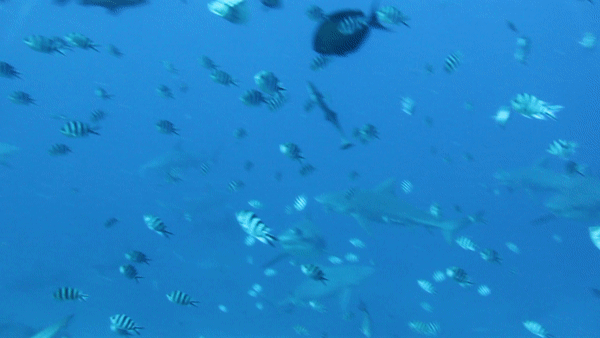

Supplement: S1 Fig — The tuna entered the water column from the top of the image, where it was immediately surrounded by several nominally herbivorous Acanthurus xanthopterus, which were observed consuming the tuna flesh. The tuna head then drew attention from several piscivorous Lutjanus bohar. At 4 seconds a large Carcharhinus leucas frightens off other fish, and consumed the tuna head whole. Throughout the video the fish are surrounded by a school of planktivorous Abudefduf sexfasciatus. (GIF) [file pone.0221781.s001.gif]
